# A case of IgG4-related cholecystitis diagnosed by transpapillary gallbladder biopsy using a novel device delivery system

**DOI:** 10.1055/a-2177-3793

**Published:** 2023-10-06

**Authors:** Yujiro Kawakami, Naohiro Kameyama, Yosuke Hirobe, Yoshiharu Masaki, Ayako Murota, Shintaro Sugita, Hiroshi Nakase

**Affiliations:** 1 Department of Gastroenterology and Hepatology, Sapporo Medical University School of Medicine, Sapporo, Japan; 2 Department of Surgical Pathology, Sapporo Medical University School of Medicine, Sapporo, Japan

A 60-year-old man who was previously diagnosed with acute cholecystitis with gallbladder stones was referred to our department for further investigation of gallbladder wall thickening. Blood tests revealed normal serum carcinoembryonic antigen (1.85 ng/mL) and carbohydrate antigen 19–9 levels (8.1 U/mL) and elevated serum immunoglobulin G4 (IgG4) levels (995 mg/dL). Abdominal ultrasonography revealed gallbladder stones and localized wall thickening of the gallbladder fundus ( Fig. 1 ). Contrast-enhanced computed tomography revealed wall thickening of both the bile duct and the gallbladder fundus ( Fig. 2 ). Endoscopic retrograde cholangiography revealed a contrast agent defect at the gallbladder fundus ( Fig. 3 ). To confirm the histological diagnosis, transpapillary gallbladder biopsy was attempted ( Video 1 ). A guidewire was inserted to help advance a newly designed endoscopic sheath (Endosheather; Piolax Medical Device, Kanagawa, Japan) into the gallbladder. Through the sheath, a targeted biopsy of the gallbladder fundus lesion was performed using biopsy forceps ( Fig. 4 ). Histopathology revealed > 10 IgG4-positive lymphoplasmacytic cells/high-power field, with an IgG4/IgG-positive cell ratio of > 40 % ( Fig. 5 ). Based on the pathological findings, we diagnosed the patient with IgG4-related cholecystitis, and we performed a laparoscopic cholecystectomy. Histological examination of the surgical specimens confirmed the gallbladder lesion as IgG4-related cholecystitis.

**Fig. 1:**
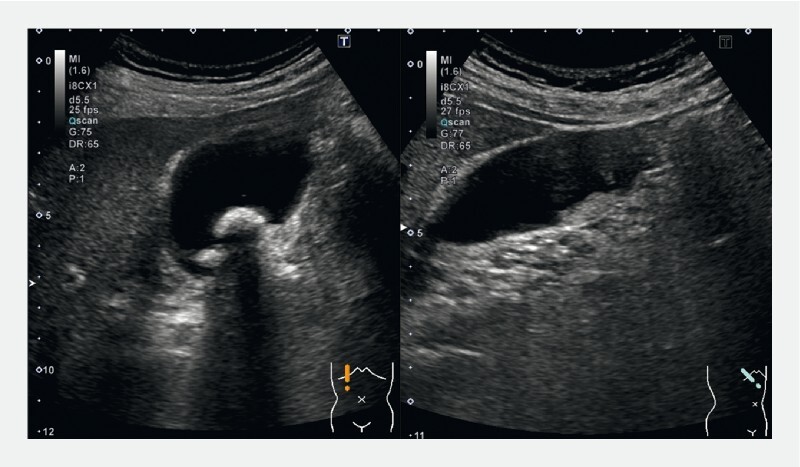
Abdominal ultrasonography revealed gallbladder stones and localized wall thickening of the gallbladder fundus.

**Fig. 2:**
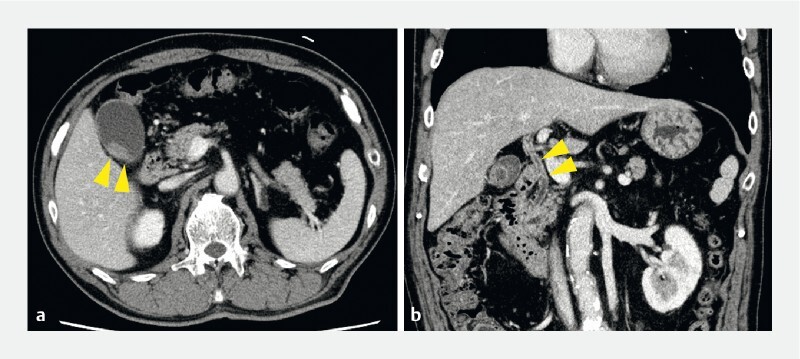
Contrast-enhanced computed tomography revealed thickening of the bile duct wall and localized wall thickening of the gallbladder fundus (arrows).

**Fig. 3:**
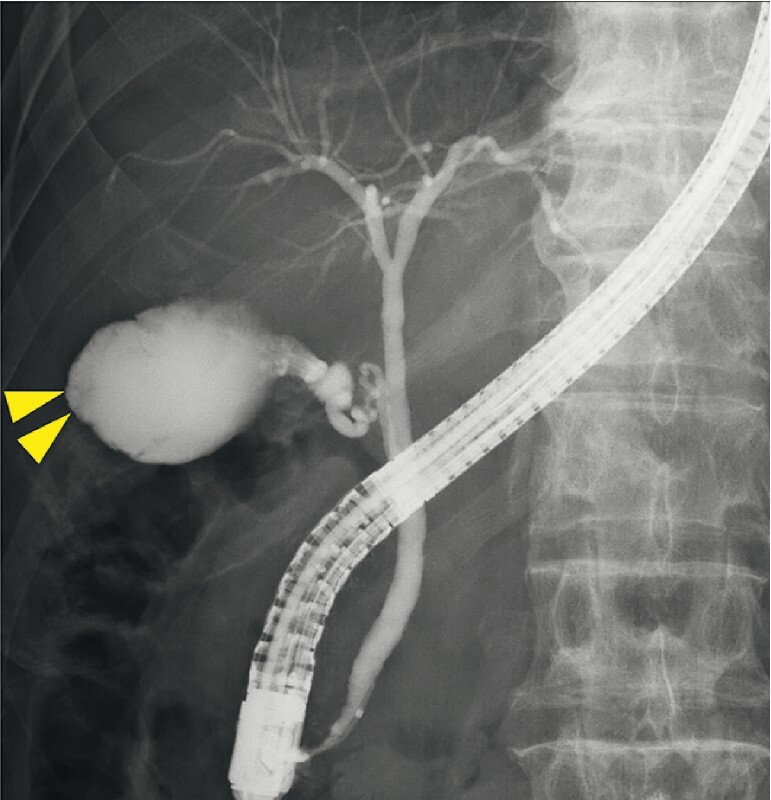
Endoscopic retrograde cholangiography revealed a contrast agent defect at the gallbladder fundus.

**Video 1** IgG4-related cholecystitis diagnosed by transpapillary gallbladder biopsy using a novel device delivery system. Source for graphical illustrations: atelier orca/Masakazu Kanzaki.


**Fig. 4:**
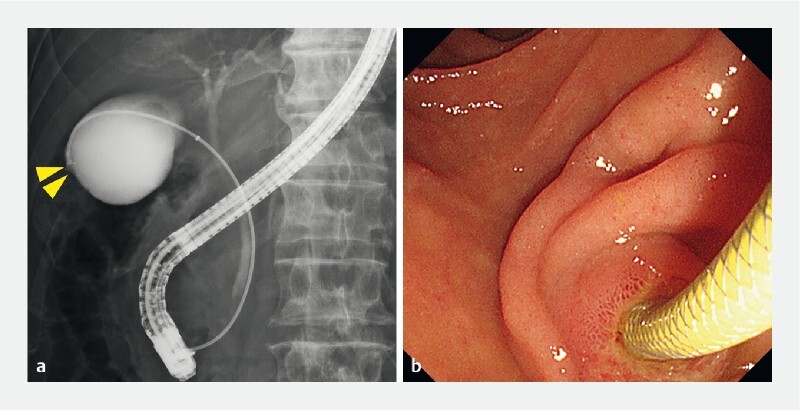
A targeted biopsy of the gallbladder fundus lesion using biopsy forceps.

**Fig. 5:**
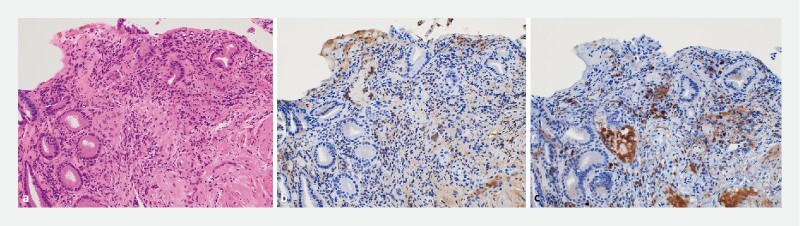
Histopathology revealed > 10 immunoglobulin G (IgG)4-positive lymphoplasmacytic cells/high-power field, with an IgG4/IgG-positive cell ratio of > 40 %. **a** Hematoxylin stain × 200. **b** IgG stain × 200. **c** IgG4 stain × 200.

IgG4-related cholecystitis is considered a lesion of IgG4-related disease 1 , usually presenting as gallbladder wall thickening and mass lesions 2 . Distinguishing between IgG4-related cholecystitis and gallbladder cancer is difficult based on imaging findings alone. As a result, surgery is often required to obtain a definitive diagnosis 3 . To the best of our knowledge, this is the first case report of IgG4-related cholecystitis diagnosed by transpapillary gallbladder biopsy.

Endoscopy_UCTN_Code_TTT_1AR_2AD

## Notes

Endoscopy E-Videos

https://eref.thieme.de/e-videos
*E-Videos* is an open access online section of the journal *Endoscopy* , reporting on interesting cases and new techniques in gastroenterological endoscopy. All papers include a high-quality video and are published with a Creative Commons CC-BY license. Endoscopy E-Videos qualify for HINARI discounts and waivers and eligibility is automatically checked during the submission process. We grant 100% waivers to articles whose corresponding authors are based in Group A countries and 50% waivers to those who are based in Group B countries as classified by Research4Life (see: https://www.research4life.org/access/eligibility/ ). This section has its own submission website at https://mc.manuscriptcentral.com/e-videos
